# Single posterior surgical management for lumbosacral tuberculosis: titanium mesh versus iliac bone graft: A retrospective case–control study

**DOI:** 10.1097/MD.0000000000009449

**Published:** 2017-12-22

**Authors:** Xin H. Yin, Zhong K. Liu, Bao R. He, Ding J. Hao

**Affiliations:** Department of Spine Surgery, Hong Hui Hospital, Xi’an Jiaotong University College of Medicine, Xi’an, China.

**Keywords:** bone fusion, iliac bone graft, lumbosacral tuberculosis, posterior debridement, posterior instrumentation, titanium mesh

## Abstract

Recently, the one-stage posterior approach for treating spinal tuberculosis (TB) has gained popularity. However, large bony defects after debridement remain a major challenge in posterior surgery. The present retrospective study aims to compare the clinical outcomes of posterior-only surgical management by titanium mesh versus iliac bone grafts for treating lumbosacral TB. This was a retrospective cohort study. From January 2006 to April 2012, 36 patients with lumbosacral TB were treated at our department. The 36 cases were divided into 2 groups: 17 patients in Group A (titanium mesh) underwent one-stage posterior internal fixation, debridement, and titanium mesh bone fusion. The 19 patients in Group B (iliac bone graft) underwent posterior instrumentation, debridement, and iliac bone graft in a single procedure. The clinical and radiographic results for the 2 groups were analyzed and compared. The mean year of patients was 49.9 ± 15.4 months in group A and 55.5 ± 12.6 months in group B. All patients were followed up for an average of 47.3 ± 8.1 months (range 36–60 months). Spinal TB was completely cured and no intraspinal infection and central nervous system complications of TB infection occurred. Bone fusion was achieved 6.4 ± 1.9 months in group A and 7.8 ± 2.1 months in group B. There was no significant statistical difference in bone fusion between the 2 groups (*P* > .05). The Oswestry Disability Index score (ODI) significantly improved between the preoperative and the last visit in either group. However, no significant difference was observed between the 2 groups at last visit (*P* > .05). There were significant differences between groups regarding the postoperative lumbosacral angle and angle correction loss at the final follow-up (*P* < .05). The average operative complication rate of Group A was less than that of Group B. Both iliac bone and titanium mesh can effectively construct anterior column defects in posterior surgery. The titanium mesh has the advantage of minor surgical invasion, effective reconstruction of large defects, and ideal sagittal alignment in lumbosacral TB for patients with osteoporosis and poor iliac bone quality.

## 1 Introduction

It is generally accepted that the aim of treating spinal tuberculosis (TB) is to debride the lesion, restore neurological function, correct spinal deformity, and enable patients to function unimpeded in their day-to-day life. A long-term cure of spinal TB lies on a stable fusion by bone grafting.^[1–3]^ Various surgical managements (for example, anterior, posterior, and combined and 2-stage approaches) have been performed on patients with lumbosacral TB.^[4–6]^ Till date, the posterior-only approach for treating spinal TB has gained popularity. This procedure has the advantage of minor surgical invasion, effective kyphosis correction, and a fewer surgical complications; whereas the reconstruction of bony defects after debridement remains a major challenge. To what extent the use of titanium mesh cages can construct the anterior column within the infected area has not been fully discussed in the posterior-only approach. To our knowledge, there is a lack of studies comparing the clinical outcomes of posterior surgical management by titanium mesh versus iliac bone grafts for lumbosacral TB. Therefore, this study aimed to review and compare the therapeutic efficacy of the 2 bone graft approaches in 36 patients with lumbosacral TB.

## 2 Methods

### 2.1 Study site and patients

This study was approved by the Hong Hui Hospital Ethics Committee. Written informed consent was obtained from all patients. We performed a retrospective review of clinical and radiographic data prospectively collected from 40 consecutive lumbosacral tubercular patients between January 2006 and April 2012. Except for 4 patients treated conservatively, the remaining (36 cases) received surgery by posterior debridement and interbody fusion. The indications for surgery were progressive neurological deficit; persistent pain because instability; severe kyphosis or kyphosis likely to progress; poor outcomes after conservative treatment. The diagnosis of lumbosacral TB was based on clinical symptoms, radiographic evidence (eg, plain radiograph, computed tomography, and magnetic resonance imaging), and both hematologic and pathological examination.^[7]^ In contrast, the excluded criteria were previous lumbosacral surgery; lumbosacral lesion induced by disease, such as metastasis or multiple myeloma.

Seventeen patients underwent single-stage internal fixation, debridement, and titanium mesh fusion to treat lumbosacral TB via a posterior approach (Group A). The remaining 19 patients underwent posterior internal fixation, debridement, and iliac bone graft fusion to treat lumbosacral TB and were labeled the control group (Group B). Details of clinical characteristics are shown in Table 1.

**Table 1:**
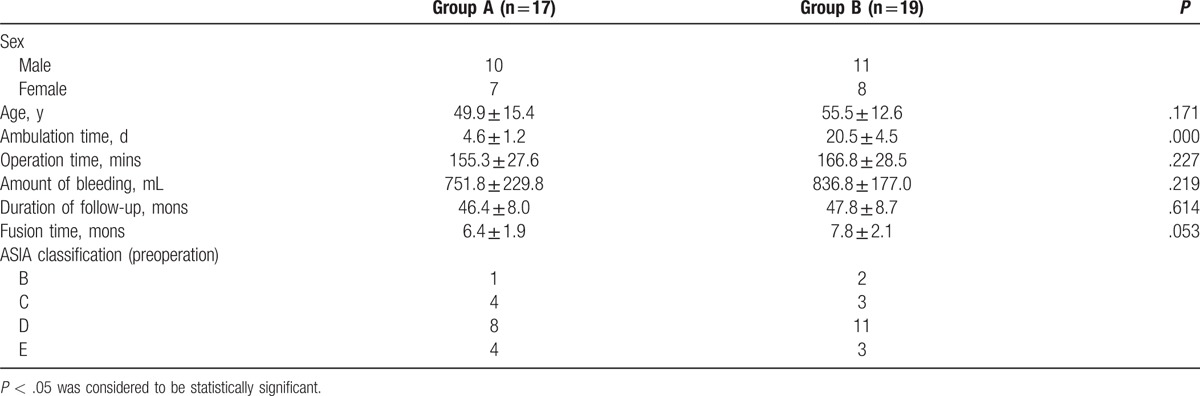
Clinical data on the patients of 2 groups.

### 2.2 Preoperative preparation

All patients were treated with HREZ chemotherapy (isoniazid 300 mg/d, rifampicin 450 mg/d, ethambutol 750 mg/d, and pyrazinamide 750 mg/d) for 2 to 4 weeks before surgery. The operation was performed when ESR and CRP gradually decreased, TB toxicity symptoms improved, and nutritional state changed improved. In general, preoperative ESRs and hemoglobin levels should not have been higher than 40 mm/h and 10 g/dL, respectively.

### 2.3 Operative technique

The patients were placed in prone position under general anesthesia with somatosensory-evoked potential monitoring. Afterward, the posterior spinal elements, including transverse processes, facet joints, and lamina were exposed (subperiosteum dissection) by extending one vertebrae above and below the involved segments via a midline incision.

According to reoperative symptoms and imaging, transpedicular screws were implanted in the side of vertebral lamina. If the upper part of the vertebrae was not destroyed by infection, the screws were also implanted in the destroyed vertebrae. A unilateral facetectomy and a laminectomy up to the medial pedicle edge were performed. A temporary rod on the offside of the nidus was stabilized to avoid nerve injury caused by spinal instability during decompression and nidus debridement. Then, the superior and inferior articular processes of the vertebrae were partially resected on the same side to expose the intervertebral space. Next, we used a flush tube to drain prevertebral abscesses. Including collapsed vertebras, the necrotic disc and cold abscesses were completely removed by curettes through the healthy bleeding bone. It should be noted that the nerve, large blood vessels, and ureter were not stretched or distracted. Correction of the lumbosacral deformity was performed by installing contoured rods and exerting compression at middle anchoring points using a cantilever bending maneuver. Collateral anterior spinal cord decompression was obtained. After thorough debridement, we formed titanium mesh in accordance with bone defects, and autogenous or allograft bone was filled with the mesh. Afterwards, the titanium mesh was implanted in the bone defect (In Group B, iliac bone was used to construct the anterior defect). Next, autologous or allograft bone particles were implanted in the lateral facet joints of diseased vertebrae and between the transverse processes. Treatment with 1.0 g streptomycin and 0.2 g isoniazid was locally administered, and drainage and incision sutures were performed postoperatively. The debrided material was sent for culturing and pathological diagnosis.

### 2.4 Postoperative procedure

The drainage tube was usually removed when drainage flow was clear and less than 30 mL/24 hours. The patients remained in bed for 14 to 28 days.^[8]^ Patients received oral HREZ chemotherapy for at least 9 months postoperatively, and isoniazid, rifampicin, and ethambutol (HRE) treatment for another 3 to 6 months. For all cases, average operation time, amount of bleeding, hospitalization, and operative complications were recorded. Clinical outcome was assessed preoperatively and at the last follow-up visit by using the Oswestry Disability Index (ODI) questionnaire.^[8]^ The preoperative and postoperative lumbosacral angle was recorded on lateral plain-film radiographs, and neurological status was recorded by the American Spinal Injury Association (ASIA) classification. Bone grafting fusion was assessed by using the radiologic criteria of Bridwell et al.^[9]^ x-Ray examination was performed every three months. All statistical analyses were conducted by using SPSS 20.0 software. A paired Student *t* test was performed to compare parameters both pre- and postoperatively and at the final follow-up. A *P* value of <.05) was considered statistically significant.

## 3 Results

Among the 36 patients, 21 were men and 15 were women, with a combined mean age of 53.4 ± 14.2 years. TB was confirmed by bacterial culture or pathological diagnosis for all 36 patients. No recurrence was noted in the 2 groups. In the iliac bone graft group, graft fracture was observed in 2 patients, whereas 3 patients occurred chronic donor site pain after surgery. ESR and CRP returned to normal levels in all patients 3 months after surgery. The complications of the TM group were significantly less than those of the iliac bone group.

All interbody fusion was thoroughly fused in the TM group, with an average fusion time of 6.4 ± 1.9 months. In the iliac bone graft group, 17/19 (89.5%) patients obtained grafted bone fusion at 7.8 ± 2.1 months. Fusion in 2 patients was delayed because of graft fracture. Although the fusion rate was higher in the TM group, no statistical significances were noted between the 2 groups (*P* > .05).

**Figure 1:**
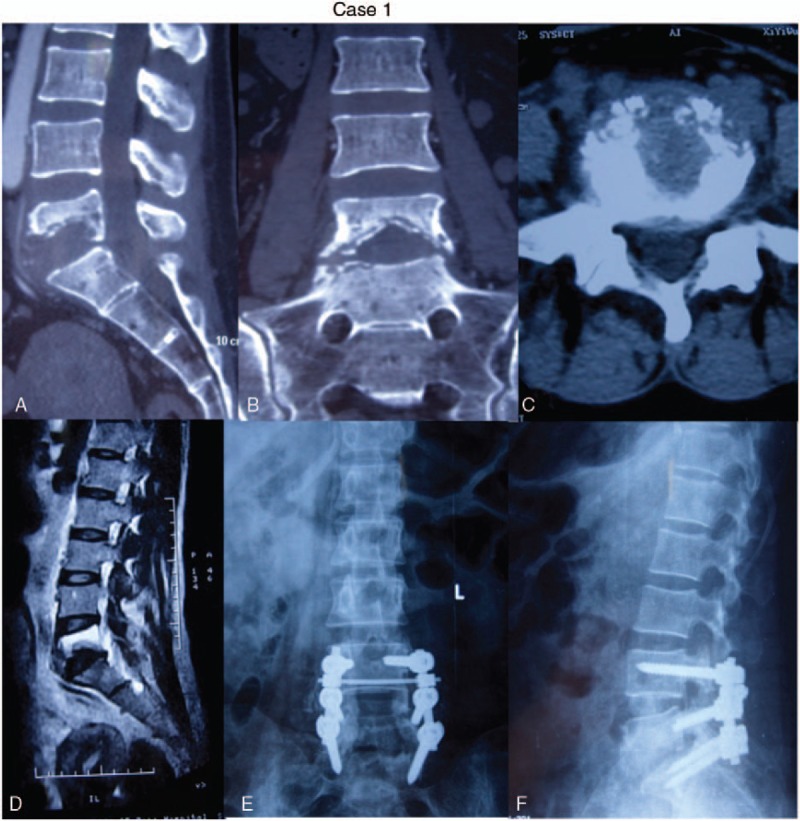
Female, 65-year, L5/S1 TB (A–D): MRI and CT showed the destruction of vertebral bodies of L5 and S1, (e,f)The lateral and anteroposterior view of x-ray showed that the anterior infected site had healed and bony union was achieved at the final follow-up.

**FIgure 2:**
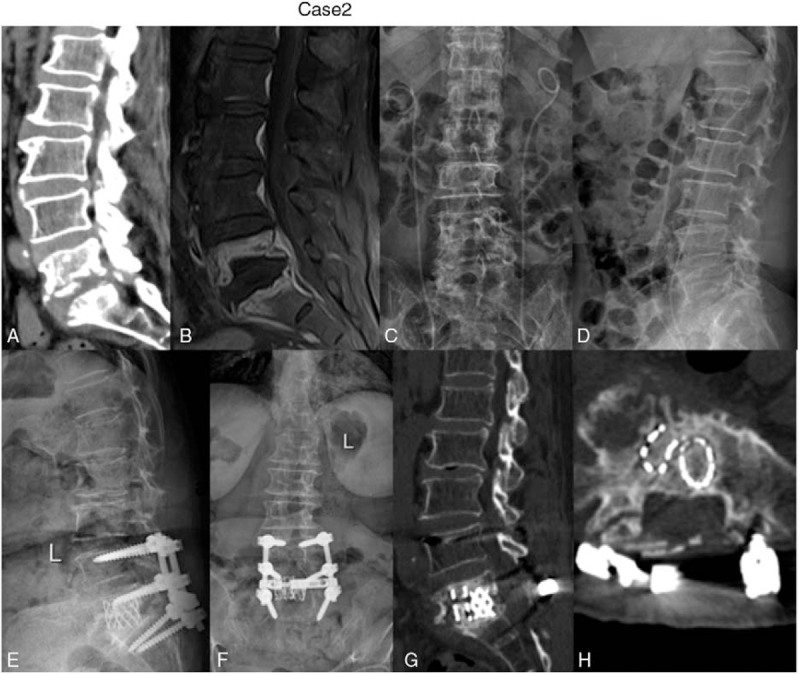
A 70-year-old female was diagnosed as having tuberculous spondylitis after an three months history of severe back pain. (A-D): x-ray, MRI, and CT showed the destruction of vertebral bodies of L5 and S1, (E–H) final follow-up radiographs showed good bone fusion.

The postoperative lumbosacral angle at final follow-up was 28.4 ± 0.9° in group A and 26.0 ± 1.5° in group B, both of which were significantly different (*P* = .001). Greater lumbosacral angle loss was observed in the iliac bone graft group compared with the TM group, which was statistically significant (*P* = 0.014). All patients were observed to significantly improve in constitutional symptoms and back pain after surgery. The mean ODI was also observed to significantly improve between the preoperative and last visit in either group, but no significant differences between the 2 groups were noted at the last visit (Table 2). In 36 patients with neurological deficit, 33 showed complete neurological recovery. Three cases with an initial B classification recovered to D. Details of the pre- and postoperative ASIA grades are given in Tables 1 and 2. The typical cases are shown in Figures 1 and 2.

**Table 2:**
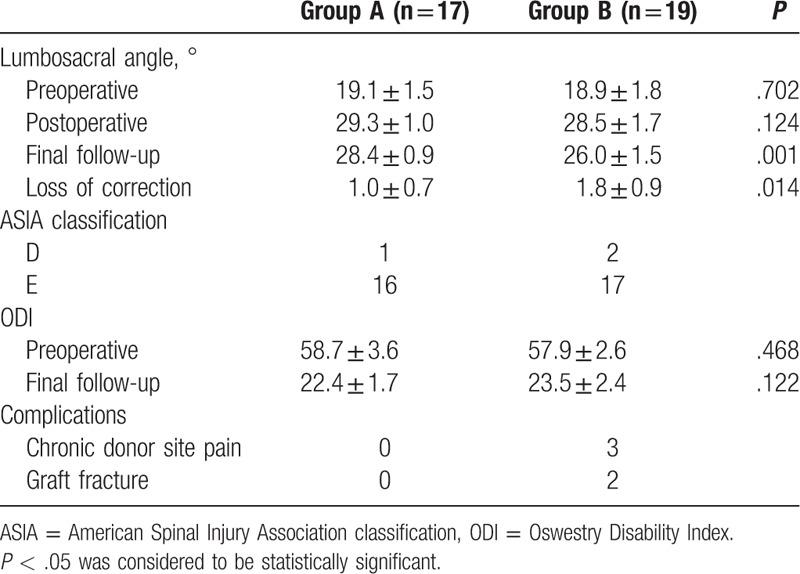
Outcomes of 2 different surgical treatments for lumbosacral tuberculosis.

## 4 Discussion

Lumbosacral TB is relatively uncommon and accounts for only 2% to 3% of all spinal TB.^[10]^ Treatment strategies for lumbosacral TB include both conservative and operative treatments. Although chemotherapy is a mainstay in the management of spinal TB, surgical treatments remain important. Surgical management of lumbosacral TB aims to completely debride the lesion by reconstructing spinal stability and restoring nerve function. Various surgical approaches have been performed on patients with lumbosacral TB. For example, those include conventional anterior-only and more minimally invasive techniques, posterior-only, and anterior–posterior approaches. Generally, the lesion is usually located in the anterior column; thus, the anterior approach is considered better for decompression and fusion. However, the procedure itself may result in the progression of kyphosis as a consequence of bone graft failure.^[11,12]^ Furthermore, owing to special anatomic characteristics and position, anterior stabilization using instrumentation is difficult to place at lumbosacral segments. Several surgeons ^[13,14]^ have advocated 2-stage surgical treatments involving anterior debridement and posterior instrumentation. Specifically, they emphasized its advantages in reaching the focal point of the disease directly and its effective decompression on the nerve. By contrast, disadvantages of the anterior–posterior approach are also apparent, with 2 positions and 2 incisions being used in the procedure. For example, a long operative time, large blood loss, large wounds, prolonged hospitalization, and comparatively higher costs makes the AP approach unsuitable for patients >65 years with a poor general state of health.^[15]^

Till date, several studies on the single-stage posterior approach for spinal TB treatment have reported good clinical efficacy.^[16–22]^ The procedure has the advantage of minor surgical invasion, effective kyphosis correction, and less complications. Nonetheless, the posterior approach allows for operations on the vertebral body at a limited angle for interbody fusion. However, given the decreased stability caused by resection of the zygapophyseal joints, the outcome of interbody fusion remains controversial. It is generally accepted that the long-term success of surgical treatment depends on a stable fusion by bone grafting. However, the reconstruction of large bony defects after debridement remains a major challenge in the single-stage posterior approach for spinal TB treatment. This is especially so for lumbosacral segments constituted by lumbar activity and biomechanical stress concentrated in junctional areas. The lumbosacral segments are unstable and tend to progress at least until bony fusion occurs. Therefore, reconstructing spinal stability is considered the most reliable evidence of the long-term success of surgical treatments.

In the previous studies, tricortical bone grafts have been used to reconstruct TB defects of the spine.^[23,24]^ Several scholars suggest that tricortical bone grafts are the gold standard for reconstructing the spine. Whereas, donor site complication rates as high as 10% have been reported after autogenous bone grafting. Chronic donor site pain has been reported in up to 40% of cases.^[25,26]^ More importantly, autograft bones are associated with fracture complications both during operation and postoperatively.^[27,28]^ In our cohort, 2 patients suffered fractures and 3 patients occurred chronic donor site pain after surgery.

Considering that titanium mesh may decrease antituberculous effectiveness, increase TB adherence, and cause TB recurrence, stabilization of the infected spine using metal implants has been controversial. Whereas, Oga et al^[29]^ evaluated the adherence capacity of mycobacterium TB to spinal instrumentation and concluded adherence was negligible, and that the use of implants in regions with active TB infection is safe. Hee et al^[30]^ found that stabilizing the spine with pedicle screws and titanium mesh in patients with tuberculous spondylitis effectively can prevent the development of kyphotic deformity, which was not observed to prevent controlling infection. Zhang et al^[31]^ reported that 28 patients with spinal TB were treated by debridement, internal fixation, and reconstruction using titanium mesh cage via a posterior-only approach. All patients obtained solid bony fusions without failure of fixation, and infections were resolved in all patients. These findings are consistent with those of our study, whereby all patients significantly improved postoperatively with regards pain. Moreover, TB was observed to fully heal. No complications related to instrumentation occurred. We affirm that the insertion of titanium mesh in tubercular infection is safe. However, a few reports have compared the clinical outcome of iliac bone graft with that of titanium mesh in posterior surgical management for lumbosacral TB. In our cohort, the titanium mesh group, an incision, no chronic donor site pain, no suffering graft fractures. In previous studies, fusion rates with titanium mesh were significantly higher than allograft bone fusion rates (95–100 vs 89.7%).^[32,33]^ In the series, all titanium meshes were thoroughly fused, with an average fusion time of 6.4 ± 1.9 months. Although the fusion rate was lower in the iliac bone graft group (89.5%), no statistical significances were observed between the two groups (*P* > .05). In our opinion, the use of a titanium cage in the treatment of lumbosacral TB offers the advantages of better sagittal balance, minor loss of correction, minor complications, and 100% to 89.5% fusion rates compared with the iliac bone graft group. From our experience, firstly, we implanted autologous bone in the diseased lateral facet joints between the transverse. Second, the titanium mesh cage could provide immediate stability, tolerate compression forces, restore disc space height, and obtain ideal sagittal alignment. Moreover, 360° interbody fusion could be conducted. In addition, we removed focal tissues and tissues in focal edges. This involved reaching the subnormal substance of bones between normal cancellous and pathologic bones, which saved the subnormal substance of bones as much as possible. It is well known that posterior transpedicular instrumentation may provide sufficient spinal stability and obviate the evolution of late angular deformity.^[34,35]^

Titanium mesh offers several significant benefits compared with the iliac bone graft. It is considered a sound option for anterior reconstruction, which eliminated the need for iliac bone harvesting. It is also helpful for patients with osteoporosis and poor iliac bone quality. We previously formed TMC in accordance with anterior column defect space after debridement, and the ideal size and height of the TMC was implanted. The formed titanium cage was observed to have a wider contact area between the cage and endplate, which resulted in a balanced force distribution more that prevented extrusion or displacement.^[36,37]^ Titanium cages offer immediate anterior column stability combined with posterior pedicle screw fixation that can tolerate compression forces.^[38,39]^ In addition, patients in the TMS group experienced immediate early ambulation with full weight beating. TMC can restore the intervertebral disc height, obtain ideal sagittal alignment, and obviation of bone graft harvesting outside the surgical site.^[40,41]^

There were some potential shortcomings of our study. For example, the number of patients reported was a few. To reaffirm the utility of this approach, a larger number of patients for our study are needed.

## 5 Conclusion

In conclusion, it has been demonstrated in our study that both iliac bone and titanium mesh can effectively construct anterior column defects in posterior surgery. However, complications and ambulation time after the titanium mesh approach were observed to be prominently a fewer than those after the iliac bone approach. This procedure has the advantage of minor surgical invasion, effective reconstruction of large defects, and ideal sagittal alignment in lumbosacral TB for patients with osteoporosis and poor iliac bone quality.
